# Burden of Intestinal Parasitic Infections and the Intensity of Helminth Infection Among Primary School Children in Sidama, Ethiopia: A Zero‐Inflated Negative Binomial Analysis

**DOI:** 10.1155/japr/2036666

**Published:** 2026-10-21

**Authors:** Aberash Eifa Dadhi, Mekdes Tigistu Yilma, Mehretu Belayneh, Ingunn Marie Stadskleiv Engebretsen, Alemselam Zebdewos Orsango

**Affiliations:** ^1^ Department of Midwifery College of Medicine and Health Science Hawassa University Hawassa Ethiopia hu.edu.et; ^2^ School of Public Health College of Medicine and Health Science Hawassa University Hawassa Ethiopia hu.edu.et; ^3^ Department of Public Health Institute of Health Sciences Wollega University Nekemte Ethiopia wollegauniversity.edu.et; ^4^ Department of Global Public Health and Primary Care Centre for International Health University of Bergen Bergen Norway uib.no

**Keywords:** helminth infection intensity, intestinal parasitic infection, protozoal infection, school children

## Abstract

**Introduction:**

Intestinal parasitic infections are a global health issue. Data on protozoal infections and the utilization of count models to describe helminth intensity are limited. This study was aimed at assessing the prevalence of intestinal parasitic infections and the intensity of helminth infections as well as its associated factors among primary school children in Shebedino District, Sidama, Ethiopia.

**Methods:**

A cross‐sectional study was conducted in 2024 involving 622 primary school children (Grade 1–2/7–10 years old) from six selected schools using a multistage stratified sampling technique. Stool samples were examined using the wet mount and Kato–Katz procedures. Logistic regression was conducted to assess factors associated with intestinal parasitic infections, and zero‐inflated negative‐binomial regression to identify factors associated with intensity of helminth infection using Stata 17.

**Result:**

A total of 622 children provided stool samples for analysis, of whom 334 (53.7%) were boys. The median age was 8.0 years (interquartile range [IQR]: 2 years). The prevalence of intestinal parasitic infections was 348 (56.0%), of whom 253 (40.7%) had a single infection. From the total parasitic infections, 311 (89.4%) were helminth, with *Ascaris lumbricoides* accounting for 302 (86.8%). Protozoal infections accounted for 110 (31.6%) of the total infections, with *G. lamblia* being the most common observed protozoa, 95 (27.3%). Males were less likely to be uninfected with *A. lumbricoides* than females (AOR = 0.6; 95% CI: 0.5, 0.7). The intensity of *A. lumbricoides* infection was significantly lower among children whose mothers had attended primary or higher education (incidence rate ratio [IRR] = 0.5; 95% CI: 0.3, 0.9).

**Conclusion:**

Intestinal parasitic infections are prevalent among primary school children in the rural Sidama region. Improving preventive measures, like access to safe water, improved food hygiene practices, and sanitation routines, while addressing proper personal hygiene through health education for school children, is urgently needed.

**Trial Registration:**

Pan African Clinical Trials Registry: PACTR202403614331028

## 1. Introduction

Intestinal parasitic infections are a major health problem in low‐resource settings.

[1]. Children from low‐income households are particularly vulnerable to intestinal parasitic infections [2] such as helminth and protozoal infections [3]. An estimated 654 million school children suffer from intestinal parasitic infections [4]. Sub‐Saharan Africa, Asia, Latin America, and the Caribbean have the highest rates of intestinal parasitic infections globally. The most prevalent helminth species are *Ascaris lumbricoides,* followed by *Trichuris trichiura* and hookworms [5, 6], whereas the most prevalent protozoal parasites are *Entamoeba histolytica,* followed by *Giardia lamblia, Balantidium coli,* and *Cryptosporidium parvum* [7, 8].

Many infected people are asymptomatic during prediagnostic stages even though the infection intensity may be light, moderate, or heavy depending on the host′s immunological status and the parasite′s virulence [9]. High infection rates and intensities disproportionately affect rural primary school children [10]. Primary school children require clean and safe drinking water, and adequate and maintained sanitation facilities including basic toilets in their homes and at school. A lack of these essential requirements may expose them to a wide range of health problems and morbidities [11].

The fecal‐oral route is a major contributor to the transmission of intestinal parasitic infections in humans, primarily through contamination of soil, feces, and polluted water sources. Personal hygiene practices are often poor due to limited access to sanitation facilities [12]. In Ethiopia, large parts of the population face inadequate water supply, sanitation, sewage systems, and safe hygiene practices, resulting in a high prevalence of intestinal parasitic infections and associated adverse health outcomes [10, 13]. The Ethiopian Federal Ministry of Health (FMOH) has instituted health education, routine deworming, and clean water, sanitation, and hygiene (WASH) programs to control and prevent intestinal parasitic infections in school children [2, 14], but implementation, supervision, and the maintenance of equipment and infrastructure remain major challenges.

The negative health impacts of intestinal parasitic infections in primary school children include undernutrition, stunting, and nutritional anemias like iron deficiency anemia, as well as related problems such as poor school attendance and lower academic performance [10, 15]. Although there is evidence of the prevalence of intestinal parasitic infections among school children in Ethiopia, their primary focus has been on soil‐transmitted helminths [14, 16]. There is limited data on protozoal infections [17], though they significantly contribute to child morbidity. Moreover, most studies have emphasized the prevalence estimates without addressing infection intensity, which is an important indicator of child morbidity. In addition, there is a scarcity of data on the use of count models to assess the intensity of helminth infections. This study is aimed at assessing the prevalence of intestinal parasitic infections, and the intensity of helminth infections as well as its associated factors among primary school children in Shebedino District, Sidama, Ethiopia.

## 2. Methodology

### 2.1. Study Design and Settings

This study was part of a school trial, and the protocol was registered on 28 March 2024 and published [18]. In order to select schools for the intervention, this school‐based cross‐sectional study was conducted from March 25, 2024, to June 30, 2024. The catchment area included Shebedino District public primary schools in the Sidama Region, one of the national regional states of Ethiopia. Leku Town (Shebedino District City Administration) is located 27 km from Hawassa, the capital city of the Sidama Region. The region is divided into 36 districts, comprising 6 urban and 30 rural districts. The rural areas of the district contain 33 primary schools. In this district, there were a total of 37,000 children registered in the primary schools. The average number of students per class was 60. Shebedino District has one general hospital, six health centers, and twenty‐three health posts [19].

### 2.2. Study Participants

Study participants were all listed and randomly selected grade one and two students, aged 7–10 years old from the six schools in the district.

### 2.3. Inclusion Criteria

Children who were grade one and two, aged 7–10 years, who provided assent, and whose parents/caregivers provided informed consent were included in the study. For children with siblings (sisters, brothers, or twins) only one was randomly selected. In cases where another sibling was randomly selected they were replaced by the next eligible student on the list.

#### 2.3.1. Exclusion Criteria

Children who did not provide assent and whose parents/caregivers did not provide informed consent were excluded from the study.

### 2.4. Sample Size Determination and Sampling Procedure

The sample size was calculated using Epi Info Version 7.2.5.0, based on factors associated with the outcome variables in large research project with multiple objectives [18]. The calculation assumed a two population proportion, a 95% confidence level, 80% statistical power, and an effect size of 2.25 where the percent of an outcome (undernutrition/wasting) in an exposed group (having intestinal parasitic infection) was 10.1%, and the percent of an outcome in the unexposed group was 21.1% [20]. Under this assumption, the largest sample size required across all outcomes in the research project was 376. After adjusting for design effect using a variance inflation factor (VIF = 1.59), assuming equal cluster sizes (interclass correlation ICC = 0.01) [21], and an average class size of 60, the corrected sample size was 598. Adding a 10% nonresponse rate, the final sample size was 658. A multistage stratified sampling technique was used to choose a representative sample of children. From Shebedino district which is selected purposively, six primary schools were selected by a simple random sampling method, and total sample size is allocated for the school depending on the number of their students. First, the number of children sampled from each school was proportional to the number of enrolled children. In the second stage, all grade one and two were included from each school, and the selection of the children was stratified by grade. In the third stage, the allocated sample size was proportionally assigned within the grades and the respective section; finally, the children were randomly selected from each section.

### 2.5. Outcome Variables

The primary outcome was the presence of intestinal parasitic infections. It was measured by a positive stool sample examination for helminth or protozoal parasites. The secondary outcome was the intensity of helminth infection.

### 2.6. Data Collection and Measurements

Data were collected using the open‐source Enketo Kobo tool by six trained teachers, two health officers and four laboratory technologists. It included a parent interview, physical examination and laboratory investigations.

Face‐to‐face interviews with parents or caregivers were held using a pretested, structured, interview‐administered questionnaire. The interview questionnaires included child′s age sex, and grade; parents′/caregivers′ education, occupation, marital status, and source of income; family size; housing condition including people per sleeping room; presence of separate cooking room; roof, wall, and floor materials; source and the time it takes to fetch water; types of latrine; and household assets (table, chair, bed with cotton mattress, mobile/fixed phone, watch, radio, television, refrigerator, computer, electricity, electric stove). It also captured ownership of domestic animals, agricultural land, car, motorcycle, bicycle, animal‐drawn cart, cooking fuels (electricity, kerosene, charcoal, biogas, wood, dried animal dung, grass/shrubs, agricultural crops); illness history of the child during 2 weeks prior to data collection; handwashing after latrine use; and deworming history of the child 6 months prior to data collection.

The physical examination of children was conducted by two trained health officers, who have a physician assistant role in Ethiopia. The examination included an overall assessment of his or her health condition with especial focus on observing children′s nails cleanliness and trimming, and footwear use [22].

To detect helminth and protozoal infections, direct microscopic examination/wet mount and Kato katz techniques were used; concentration or antigen‐based method to detect protozoal infections was not conducted. Fresh stool samples from children were collected after trained medical laboratory professionals provided the children with the necessary information and guidance with the assistance of their parents. Each child got a labeled, clean, dry, and leak‐proof stool cup. Thereafter, within 30 min of sample collection, the direct microscopic examination, or wet mount, was conducted using approximately 5 mg (the size of a match head) of feces mixed with two drops of normal saline placed on a clean slide by following standard laboratory procedures [14].

For the Kato–Katz technique, approximately 2 g of feces were sieved through a nylon sieve. From the sieved feces, 41.7 mg was used to determine the number of eggs and helminth species observed under a light microscope, following standard laboratory procedures [23]. The number of eggs counted was multiplied by a factor of 24 to obtain the number of eggs per gram (EPG), as recommended by the World Health Organization [24].

### 2.7. Data Quality Control

Data collectors received 2 days of training on the study objectives, data collection methods and procedures, sample collection and transportation, and quality control mechanisms. Data collectors comprised six school teachers with bachelor′s degree, two health officers, and four laboratory technologists. In addition, two experienced supervisors (one nurse and one laboratory technologist) oversaw the data collection process. The interview questionnaire was prepared in English, translated into the local language (Sidaamu Afoo) and back translated to English to check for inconsistences. A pretest was conducted on 5% of the study sample (34 children), and necessary amendments to the data collection tool and procedures were made before the actual data collection. To check the functionality of the microscope as well as the accuracy of the laboratory professional engaged in conducting the study, negative and positive control slides were used daily. The slides were examined independently by two medical laboratory technologists. Any discordance in the results was resolved by a third laboratory professional (supervisor).

### 2.8. Data Management and Analysis

The data were downloaded to Excel and exported to the statistical software SPSS Version 27 for data cleaning and further coding. Stata Version 17 was used for data analysis. The descriptive results were expressed as means and standard deviations (SD), median and interquartile range (IQR) for continuous variables, frequencies, and percentages for categorical variables.

The wealth index was computed using principal component analysis (PCA) in SPSS Version 27. It was based on 19 variables meeting the inclusion criteria (< 95% and > 5% frequency). These included household conditions (people per sleeping room, separate cooking room, roof and floor materials, source of drinking water, type of latrine) and household assets (table, chair, bed with cotton mattress, phone, watch, radio, cooking fuels [electricity, wood, dried animal dung, grass/shrubs, agricultural crop]), ownership of domestic animals and agricultural land. First, we changed all the variables into binary as “Yes” (present) that was coded as “1” or “No” (absent) that was coded as “0.” Following all the steps in PCA, the Kaiser–Meyer–Olkin (KMO) sampling adequacy test was 56.3% with a significance of (< 0.001) Bartlett′s test of sphericity. Only six elements, the material of the floor, cooking fuel (grass/shrubs), and source of drinking water, owner of radio, table and chair had an eigenvalue higher than one; the total variance explained by the first variable was 31%. The first explanatory variable was categorized into three tertiles: poor, medium, and least poor considering that the majority were relatively poor in the area [25–28].

A chi‐square test was performed to explore any statistical relationship between study participants′ characteristics and the outcome variables. Bivariable logistic regression was used to predict the likelihood of being infected and those variables with a *p* value less than 0.25 were eligible for the multivariable logistic regression modelling (Table S1). Multicollinearity among the variables was checked using the variance inflation factor, where all the covariate values were less than five. Multivariable logistic regression was conducted to identify possible confounders. Variables with *p* value less than 0.05 were considered statistically significant.

The intensity of infection was classified according to the WHO guideline for thresholds: for *A. lumbricoides*, light infection (1–4999 EPG), moderate (5000–49,999 EPG), and heavy (greater than 50,000 EPG); for *T. trichiura* and hookworm, light (1–1999 EPG), moderate (2000–3999 EPG), and heavy (greater than 4000 EPG); whereas for *Hymenolepis nana* (*H. nana*) and *Schistosoma mansoni* (*S. mansoni*), light (1–99 EPG), moderate (100–399 EPG) and heavy (greater than 400 EPG) [29].

To identify potential factors that were associated with infection intensity, a count model was applied using fecal egg counts for the most prevalent helminth infection, *A. lumbricoides*. A Poisson regression model is appropriate for count data; however, the assumption of equal dispersion was violated as indicated by the mean EPG of *A. lumbricoides* (5602.3) being substantially lower than the variance (15,963.9). Moreover, the distribution included a large number of zero counts. To address the excess zero (median = 0), we employed a zero‐inflated negative binomial (ZINB) regression. Even though the model selection was not supported by the Vuong test (*Z* = 0.02; *p* = 0.4937), we relied on the theoretical distribution of the outcome, the overdispersion alpha indicator, and Akaike information criterion (AIC) and Bayesian information criterion (BIC) fit statistics. The ZINB model demonstrated a superior fit with AIC = 6373.681 and BIC = 6474.502 over the negative binomial (NB) model AIC = 6481.493 and BIC = 6534.095 and a substantial reduction in the overdispersion parameter (Alpha) in the ZINB model was 3.10 compared with that of the NB model which was 15.0 (Table S2 and Table S3), justifying its selection. The ZINB model consisted of two components: binary outcome model (infected vs. uninfected [zero]) and a count model (infection intensity). The count model included the following variables selected based on factors identified as likely to predict intensity: child′s sex and grade, maternal educational status, occupation of the father, wealth index, child walking barefoot, handwashing practice after latrine use, illness history 2 weeks prior to data collection, people per sleeping room, and separate cooking room. The zero‐inflated model component of the model included the same set of variables. To account for the clustering, the regression models were estimated using cluster‐robust standard errors with clustering at the school level. Because the study included only six schools, we conducted a bootstrap procedure to address the small number of clusters, which may otherwise have compromised the robustness of statistical inference. The school was used as the clustering unit; and 64 bootstrap replications with random number seed of 999 were applied to obtain bootstrap statistical inference [30]. Bootstrap results are reported in our main findings, and for the purpose of transparency, multivariable logistic regression and ZINB models based on conventional cluster‐robust standard errors are presented in the Supporting Information (Tables S4 and S5).

## 3. Results

### 3.1. Socioeconomic and Demographic Characteristics

From a total of 658 study participants, 622 had complete data in interviews, physical examinations, and stool samples for analysis, providing a 94.5% response rate. From those who completed the study, 334 (53.7%) were males; the median age of the children was 8.0 years (IQR: 2 years). Most respondents, 438 (70.4%) were mothers followed by fathers, 168 (27.0%) and other caregivers, 16 (2.6%). The median (IQR) minutes required to fetch drinking water from its source to home was 40 (74). Almost all parents, 603 (97.0%) were married and regarding to occupational status, 575 (92.4%) of the mothers were housewives and 425 (68.3%) of fathers were farmers (Table 1).

**Table 1:** Socioeconomic and demographic characteristics of the parents and children in relation to intestinal parasitic infections in Sidama, Ethiopia, year 2024 (*N* = 622).

Variable	Options	Intestinal parasitic infections		Total
		Positive *n* = 348	Negative *n* = 274	
Sex	Male	203 (58.3)	131 (47.8)	334 (53.7)
	Female	145 (41.7)	143 (52.2)	288 (46.3)
Grade	Grade one	201 (57.8)	123 (44.9)	324 (52.1)
	Grade two	147 (42.2)	151 (55.1)	298 (47.9)
Mother′s education	No formal education	101 (29.0)	59 (21.5)	160 (25.7)
	Primary	216 (62.1)	185 (67.5)	401 (64.5)
	Secondary and above	29 (8.3)	27 (9.9)	56 (9.0)
	Missing	2 (0.6)	3 (1.1)	5 (0.8)
Father′s education	No formal education	59 (16.9)	30 (11.0)	89 (14.3)
	Primary	225 (64.7)	189 (69.0)	414 (66.5)
	Secondary and above	48 (13.8)	45 (16.4)	93 (15.0)
	Missing	16 (4.6)	10 (3.6))	26 (4.2)
Occupation of father	Farmer	215 (61.8)	210 (76.6)	425 (68.3)
	Government/nongovernmental organization employer	7 (2.0)	11 (4.0)	18 (2.9)
	Daily labor	70 (20.1)	27 (9.9)	97 (15.6)
	Merchant/others	40 (11.5)	16 (5.8)	56 (9.0)
	Missing	16 (4.6)	10 (3.7)	26 (4.2)
Occupation of the mother	Housewife	319 (91.7)	256 (93.4)	575 (92.4)
	Merchant/daily labor	19 (5.4)	9 (3.3)	28 (4.5)
	Farmer/others	8 (2.3)	6 (2.2)	14 (2.3)
	Missing	2 (0.6)	3 (1.1)	5 (0.8)
Marital status of the parents	Married	338 (97.1)	265 (96.7)	603 (97.0)
	Single	4 (1.2)	6 (2.2)	10 (1.6)
	Others	6 (1.7)	3 (1.1)	9 (1.4)
Family size	< 5	90 (25.9%)	68 (24.8)	158 (25.4)
	≥ 5	258 (74.1)	206 (75.2)	464 (74.6)
Number of people per sleeping room	< 5	294 (84.5)	243 (88.7)	537 (86.3)
	≥ 5	54 (15.5)	31 (11.3)	85 (13.7)
Separate cooking room	Yes	171 (49.1)	159 (58.0)	330 (53.0)
	No	177 (50.9)	115 (42.0)	292 (47.0)
Domestic animals ownership	Yes	103 (29.6)	89 (32.5)	192 (30.9)
	No	245 (70.4)	185 (67.5)	430 (69.1)
Source of income	Agriculture	221 (63.5)	218 (79.6)	439 (70.6)
	Daily labor	79 (22.7)	31 (11.3)	110 (17.7)
	Trade	26 (7.5)	14 (5.1)	40 (6.4)
	Others	22 (6.3)	11 (4.0)	33 (5.3)
Wealth index	Poor	129 (37.1)	94 (34.3)	223 (35.8)
	Medium	156 (44.8)	112 (40.9)	268 (43.1)
	Least poor	63 (18.1)	68 (24.8)	131 (21.1)
Source of water	Tap water	227 (65.2)	188 (68.6)	415 (66.7)
	Protected well	99 (28.5)	44 (16.1)	143 (23.0)
	Surface water/other	22 (6.3)	42 (15.3)	64 (10.3)
Type of latrine	No latrine/open field	26 (7.5)	9 (3.3)	35 (5.6)
	Open pit	299 (85.9)	235 (85.8)	534 (85.9)
	Pit latrine with slab	23 (6.6)	30 (10.9)	53 (8.5)

### 3.2. Health Status and Hygiene Characteristics of Primary School Children

Among children with complete data, 109 (17.5%) had a history of illness within 2 weeks prior to data collection. The identified illnesses were diarrhea and vomiting, fever, and cough. Among these children 68 (62.4%) had a history of only one of the three illnesses, and 10 (9.2%) had a history of all the three illnesses. Among all children, about two thirds of the children had dirty and uncut nails 360 (57.9%) (Table 2).

**Table 2:** Health status and hygiene characteristics of primary school children in relation to intestinal parasitic infections Sidama, Ethiopia, year 2024 (*N* = 622).

Variable	Options	Intestinal parasitic infections		Total
		Positive *n* = 348	Negative *n* = 274	
Illness history within 2 weeks prior to data collection	Yes	70 (20.1)	39 (14.2)	109 (17.5)
	No	278 (79.9)	235 (85.8)	344 (82.5)
History of diarrhea and vomiting	Yes	29 (8.3)	21 (7.7)	50 (8.0)
	No	319 (91.7)	253 (92.3)	572 (92.0)
History of fever	Yes	34 (9.8)	18 (6.6)	52 (8.4)
	No	314 (90.2)	256 (93.4)	570 (91.6)
History of cough	Yes	39 (11.2)	19 (6.9%)	58 (9.3)
	No	309 (88.8)	255 (93.1)	564 (90.7)
Child′s nail condition	Clean and cut nails	37 (10.6)	39 (14.2)	76 (12.2)
	Dirty and cut nails	88 (25.3)	58 (21.2)	146 (23.5)
	Clean and uncut nails	14 (4.0)	20 (7.3)	34 (5.5)
	Dirty and uncut nails	205 (58.9)	155 (56.6)	360 (57.9)
	Missing	4 (1.2)	2 (0.7)	6 (0.9)
Child washing hands after latrine use	Yes	303 (87.1)	264 (96.3)	567 (91.2)
	No	45 (12.9)	10 (3.7)	55 (8.8)
Child walking barefoot	Yes	52 (14.9)	21 (7.7)	73 (11.7)
	No	296 (85.1)	253 (92.3)	549 (88.3)
Child received deworming tablet within the last 6 months	Yes	275 (79.0)	219 (79.9)	494 (79.4)
	No	73 (21.0)	55 (20.1)	128 (20.6)

### 3.3. Magnitude of Intestinal Parasitic Infections

The overall prevalence of intestinal parasitic infections was 348 (56.0%) (95% CI: 52.0, 60.0). The prevalence of intestinal parasitic infection was 203 (58.3%) among male children, 201 (57.8%) among grade one children (Table 1) and 275 (79.0%) among children who reported they received a deworming tablet within the last 6 month of data collection (Table 2).

From the observed intestinal parasitic infections (*n* = 348), helminth infection accounted for 311 (89.4%); among these, *A. lumbricoides* was the most common helminth infection and accounted for 302 (86.7%). In addition, protozoal infections accounted for 110 (31.6%) of the infected cases. *G. lamblia* was the leading protozoal infection (95 [27.3%]), followed by *E. histolytica/dispar* (15 [4.3%]). Others helminth 7 (2.0%) in Figure 1; includes (*S. mansoni* 1 (0.3%), *Enterobius vermicularis (E. vermicularis)* 2 (0.6%), and *Taenia* species 4 (1.1%) (Figure 1).

**Figure 1:**
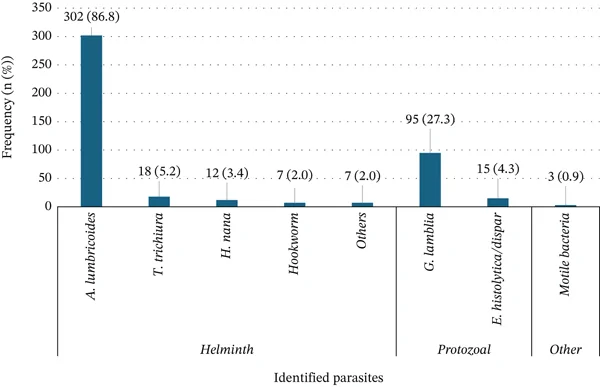
Distribution (*n* [%]) of the types of parasitic infection among school children having parasitic infection, Sidama, Ethiopia, year 2024 (*n* = 348).

### 3.4. Mixed Intestinal Parasitic Infection

From the total study participants (*n* = 622) 253 (40.6%) of children were infected with a single parasite, 83 (13.3%) were infected with double‐parasitic infections, whereas 12 (1.9%) were infected by tri‐ and quadri‐parasitic infection. For those with single infection, helminth infection alone accounted for 240 (38.5%) of cases, whereas protozoal infection alone accounted for 37 (5.9%) of cases.

Multiparasitic infections were identified in 95 (15.2%), among these helminth‐protozoal coinfection was the most common combination, occurring in 68 (10.9%) of children. Helminth‐helminth coinfection was found in 24 (3.8%), whereas helminth infection with motile bacterial infection was identified in 3 (0.5%) (Figure 2).

**Figure 2:**
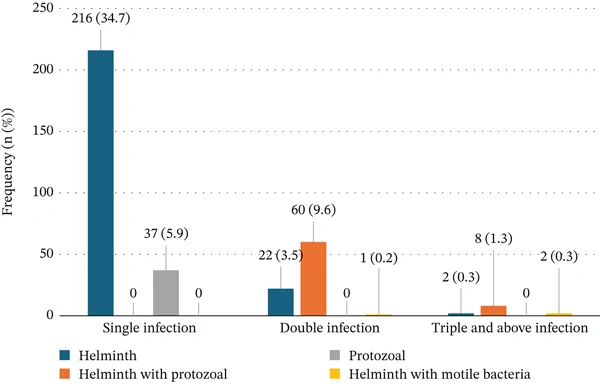
Distribution of number of parasitic infection among primary school children, Sidama, Ethiopia, year 2024 (*n* = 622).

### 3.5. Intensity of Helminth Infection

The intensity of helminth infections ranged from light to heavy. From children who were infected with *A. lumbricoides*, 194 (64%) were light, 87 (28.8%) of them had moderate, and 21 (7%) had heavy intensity of the infection (Table 3).

**Table 3:** Intensity of helminth infections among primary school children in Sidama, Ethiopia, year 2024 (*n* = 311).

Parasite species	Infection intensity	Frequency	
		*n*	%
*A. lumbricoides*	Light	194	64.2
	Moderate	87	28.8
	Heavy	21	7.0
*H. nana*	Light	11	3.5
*T. trichiura*	Light	16	5.1
	Heavy	2	0.6
Hookworm	Light	7	2.3
*S. mansoni*	Light	1	0.3
*Taenia species*	Light	4	1.3
*E. vermicularis*	NA	2	0.6

*Note:* NA: The egg count was missed for this specific parasite.

### 3.6. Factors Associated With Prevalence of Intestinal Parasitic Infections

Male children were more likely to be infected with intestinal parasitic infections compared with female (AOR = 1.5; 95% CI: 1.2, 1.8). Grade one students were more likely to be infected with intestinal parasitic infections compared with grade two students (AOR = 1.5; 95% CI: 1.1, 2.1). Children whose father′s occupation was other than farmer was more likely to be infected with intestinal parasitic infection than children whose father′s occupation was farmer (AOR = 2.1; 95% CI: 1.3, 3.3) (Table 4).

**Table 4:** Factors associated with intestinal parasitic infections among primary school children, Sidama, year 2024 (*N* = 622).

Variables	Options	Intestinal parasitic infections		COR (95% CI)	AOR (95% CI)
		Positive *n* = 348	Negative *n* = 274		
Sex	Male	203 (58.3)	131 (47.8)	1.5 (1.3, 1.8)	1.5 (1.2, 1.8) ^∗^
	Female	145 (41.7)	143 (52.2)	1	1
Grade	One	201 (57.8)	123 (44.9)	1.7 (1.2, 2.4)	1.5 (1.1, 2.1) ^∗^
	Two	147 (42.2)	151 (55.1)	1	1
Educational status of mothers	Primary+	245 (70.8)	212 (78.2)	0.7 (0.4, 1.0)	0.8 (0.6, 1.1)
	No formal education	101 (29.2)	59 (21.8)	1	1
Occupation of the father	Other than farmer	117 (35.2)	54 (20.4)	2.1 (1.4, 3.2)	2.1 (1.3, 3.3) ^∗^
	Farmer	215 (64.8)	210 (79.6)	1	1
Wealth index	Poor	133 (38.2)	77 (28.1)	1.5 (0.7, 3.3)	1.0 (0.4, 2.2)
	Medium	156 (44.8)	112 (40.9)	1.5 (0.9, 2.4)	1.2 (0.8, 1.7)
	Least poor	63 (18.1)	68 (24.8)	1	1
Walking barefoot	Yes	52 (14.9)	21 (7.7)	2.1 (0.9, 4.8)	1.4 (0.7, 2.8)
	No	296 (85.1)	253 (92.3)	1	1
Illness history 2 weeks prior to data collection	Yes	70 (20.1)	39 (14.2)	1.5 (1.1, 2.3)	1.2 (0.8, 1.9)
	No	278 (79.9)	235 (85.8)	1	1
Separate cooking room	No	177 (50.9)	115 (42.0)	1.4 (1.1, 1.9)	1.2 (0.9, 1.7)
	Yes	171 (49.1)	159 (58.0)	1	1

*Note:* Educational status primary+ = (primary, secondary, and above), Occupation other than farmer = (daily laborer, government employer/nongovernmental organization, merchant, or other). This is a bootstrap report.

Abbreviations: AOR, adjusted odds ratio; COR, crude odds ratio.

^∗^ *p* value < 0.05.

### 3.7. Factors Associated With Intensity of *A. lumbricoides*: A ZINB Regression

Males were less likely to be uninfected with *A. lumbricoides* than females (AOR = 0.6; 95% CI: 0.5, 0.7). Grade one children were less likely to be uninfected with *A. lumbricoides* than grade two children (AOR = 0.7; 95% CI: 0.6, 0.9). Children of mothers with primary and above educational status had lower infection intensity compared with those whose mothers had no formal education (IRR = 0.5; 95% CI: 0.3, 0.9). Children whose fathers had occupations other than farmer were less likely to be uninfected with *A. lumbricoides* (AOR = 0.6; 95% CI: 0.4, 0.9) and had higher infection intensity compared with those whose fathers were farmers (IRR = 1.7; 95% CI: 1.2, 2.3) (Table 5).

**Table 5:** Zero‐inflated negative binomial regression model for *A. lumbricoides* fecal egg count among primary school children in Sidama, Ethiopia, year 2024 (*N* = 622).

Variables	Options	Zero‐inflated negative binomial	
		Negative binomial	Zero‐inflated
		Infection intensity IRR (95% CI)	Infection probability AOR (95% CI)
Sex	Male	1.2 (0.8, 1.8)	0.6 (0.5, 0.7) ^∗^
	Female	1	1
Grade	One	1.5 (1.0, 2.2) ^∗^	0.7 (0.6, 0.9) ^∗^
	Two	1	1
Educational status of mothers	Primary+	0.5 (0.3, 0.9) ^∗^	1.4 (1.0, 2.0)
	No formal education	1	1
Occupation of fathers	Other than farmer	1.7 (1.2, 2.3) ^∗^	0.6 (0.4, 0.9) ^∗^
	Farmer	1	1
Wealth index	Poor	0.8 (0.5, 1.2)	1.0 (0.4, 2.6)
	Medium	1.1 (0.8, 1.5)	0.8 (0.5, 1.3)
	Least poor	1	1
Walking barefoot	Yes	1.2 (0.7, 2.0)	0.7 (0.3, 1.5)
	No	1	1
Illness history 2 weeks prior to data collection	Yes	1.5 (0.8, 2.7)	0.9 (0.5, 1.4)
	No	1	1
People per sleeping room	≥ 5	1.3 (0.7, 2.5)	0.9 (0.4, 2.2)
	< 5	1	1
Separate cooking room	No	0.9 (0.4, 2.0)	1.0 (0.7, 1.4)
	Yes	1	1

*Note:* This is a bootstrap report.

Abbreviations: AOR, adjusted odds ratio; CI, confidence interval; IRR, incidence rate ratio.

^∗^ *p* value < 0.05.

## 4. Discussion

In this study, 56.0% of the primary school children were infected with intestinal parasitic infections, signifying a major public health problem. *A. lumbricoides* was the most frequently detected helminth, followed by *T. trichiura*, whereas *G. lamblia* was the most prevalent protozoal infection, highlighting a diverse range of intestinal parasitic infections among the primary school children. Almost three‐fourth of the children had a single parasitic infection, whereas a quarter had multiple parasitic infections. This study found a substantial association between intestinal parasitic infections and the child′s sex and grade level. The intensity of *A. lumbricoides* infection decreased among children whose mothers had primary or higher education and increased among children who did not practice handwashing after latrine use and those whose fathers′ occupation was other than farming. These findings indicate that behavioral and demographic factors are important associated factors with the intensity of *A. lumbricoides* infection.

In our study, the overall prevalence of intestinal parasitic infections was 56.0%. This finding is consistent with a school based cross‐sectional study conducted Debre Berhan town, 52.9% [31], in Gedio zone of Ethiopia, 56% [17], in Hawassa town, 59.8% [32], and a school based cross‐sectional study conducted in Tanzania, 56.2% [33] and close to systematic review and meta‐analysis conducted in Colombia, 66%; with an overlapping confidence interval [34], whereas, it is higher than that reported in school based cross‐sectional studies conducted in Northwest Ethiopia, 26.8% and 49.% [9, 35], Southern Ethiopia, 21.7% [16]; and rural schools of Nepal, 44% [36], and systematic review and meta‐analysis study conducted in Turkey, 29% [37]. The prevalence in our study was lower than that reported in a school based cross‐sectional study conducted in the South Gonder Zone of Ethiopia, which was 65.4% [13]. Furthermore, it is substantially lower than the one reported in school based cross‐sectional studies conducted in Pakistan, 82% [38], and Northwestern Tanzania, 90.6% [39]. These disparities could be due to differences in environmental, socioeconomic, health system conditions, seasonal variation, and differences in the methods of diagnosis.

Among the total study participants, 40.6% had single parasitic infections, whereas the remaining 15.2% had multiparasitic infections. This finding is in line with study conducted in Hawassa, 36.6% of the participants had a single infection, and 23.1% had multiparasitic infections with an overlapping confidence interval [32]. In contrast, in a school‐based cross‐sectional study conducted in Gojam, 23.2% of the participants had a single infection, and 3.6% had multiparasitic infections [9]. These differences might be related to variation in socioenvironmental conditions, source of water, personal hygiene, and differences in deworming coverage and time. In our study, the most identified helminth was *A. lumbricoides* 86.8%. Protozoal infections were detected in 31.6% of primary school children, with *G. lamblia* accounting for 27.3% of the protozoal cases. This finding is in line with a school‐based cross‐sectional study conducted in Hawassa [32] and a study conducted in Debre Berhan town [31], as well as a study conducted in Pakistan [38]. These figures differ from those reported in a school‐based cross‐sectional study conducted in Harbu Tulu, northeast of Ethiopia, and the North Shewa Zone of Central Ethiopia [40], where the leading parasite was *E. histolytica/dispar* at 8.3% [15] and in study conducted in Northwest Ethiopia where predominant parasite was hookworm 22.2% [35] and a study conducted in Nigeria where the predominant parasite was *Strongloides stercoralis* [41]. This difference might be due to differences in the route of transmission, as *A. lumbricoides* is primarily a soil‐transmitted helminth, whereas *E. histolytica/dispar* and *G. lamblia* are mostly associated with unsafe water and poor food hygiene practices. Hookworm mostly transmitted through skin penetration [42], and children who walk barefoot are more exposed to this infection. These disparities could also be related to geographic and ecological variations that determine species‐specific transmission dynamics. This might have implications for treatment and control strategies [8]. Ideally, a nationwide mapping of interventions coverage and WASH conditions, combined with data on the burden of infection, could shed light on real‐life effectiveness and gaps in services. In our study the prevalence of deworming in the last 6 months was high, which was approximately 80%, and in contrast, the prevalence of intestinal parasitic infection was 56%. Possible explanations for this disparity include rapid reinfection, incomplete treatment coverage, variable drug effectiveness, or recall bias in reporting deworming history [43]. In general deworming lowers intensity of infection and morbidity, but does not prevent reinfection unless environmental and biological variables are addressed.

Male children were more likely to have intestinal parasitic infections than female children. This result is consistent with school based cross‐sectional studies carried out at Harbu Tulu [15], the Northcentral Region of Ethiopia [44], as well as a school based cross‐sectional study conducted in Kenya [45]. This may be attributed to cultural practices in which males are more likely to engage in outdoor activities such as playing football or herding cattle, whereas females are more likely to engage in activities such as caring for younger siblings, carrying water, and cooking, which could result in differences in exposure to contamination.

Intestinal parasitic infection was more common among grade one students than grade two students. This finding is consistent with a school‐based cross‐sectional study conducted in Northeastern Ethiopia [31] and Tanzania [39]. The higher prevalence among younger children may be attributed to greater exposure to contaminated environments, as they are more likely to play with soil and have less developed hygiene practices. Additionally, it may reflect a gap in health programs between the infant and young child service period and the school health services that provide deworming.

Regarding infection intensity, most infections were classified under light intensity, but that of the *A. lumbricoides* ranged from light to heavy, and of the *T. trichiura* ranged from light to moderate, consistent with a school based cross‐sectional study carried out in Gedio, Ethiopia [17]. However, school based cross‐sectional studies carried out in Northcentral Ethiopia and Afghanistan reported only light infection intensity, which contrasts with our finding on *A. lumbricoides* and *T. trichiura* [4, 46]. This difference suggests that, although overall transmission levels may be comparable, children in our study area may be at risk of repeated or inadequately treated infection leading to higher parasite burdens. Variation in deworming coverage, access to safe and clean water, hygiene behaviors, frequency of mass deworming, and school health support services could partly explain the presence of moderate and severe infections in our setting. According to our ZINB model, the intensity of *A. lumbricoides* infection was lower among children whose mothers had primary or higher education. Males and grade one children had lower odds of being uninfected with *A. lumbricoides*. This finding is comparable to another study conducted in Ethiopia [17]. This shows that certain socioeconomic factors such as education and hygiene behaviors have a major influence on the intensity of *A. lumbricoides* infection among school children.

The intensity of *A. lumbricoides* was higher among children whose fathers′ occupation were other than farming. This finding is most likely due to unstable economic income or irregular routines. The other reason might be if a father had unstable occupation like daily laborer, they may be mobile from place to place, which might increase the risk of bringing infection to home and increase the risk of child′s infection. Furthermore, those fathers who engaged in nonfarming activities might have less time to supervise their children which might reduce the care they could provide for their children. Since these factors might not be a direct contributing factor for the outcome, they must be interpreted cautiously.

## 5. Strength and Limitation of the Study

The use of the Kato–Katz method and a count model to describe helminth intensity was among the key strengths of this study. This study would have been stronger if it had compared both rural and urban schools. An inherent limitation of cross‐sectional studies is the risk of reverse causality; that is, the infections themselves could influence some of the observed behaviors; however, this is considered unlikely based on current understanding of these infections. That said, the effects of the infections were a bit unclear as the fever and cough were more frequent in infected children, but not their overall recent morbidity. This may indicate some level of immune responses to the stress of being infected, but could also indicate increased susceptibility to parasite infections given other infections. Adhering to the established understanding of risk factors for infections, we excluded sickness symptoms from the models. Moreover, multiple stool sample analyses would have increased the detection rate of infection. In contrast, in our case we used single stool sample for feasibility reasons; this might underestimate the prevalence of intestinal parasitic infection and egg count. However, we used a two stool sample analysis technique on a single stool sample, which may enhance the detection rate. In addition, for protozoal infection detection no additional confirmatory or concentration technique was used. Furthermore, recall bias in self‐reported behavioral data as well as bias in subjective observations may have affected the findings. We also acknowledged that cluster‐robust variance estimators perform better with a large number of clusters and have noted this as one of the limitations of this study; to handle this, a bootstrap test was conducted and bootstrap statistical inference is reported in the result. Finally, this study did not consider seasonal variation, which is recommended.

## 6. Conclusions

Intestinal parasitic infections are a major public health problem among school children in rural Sidama Region. Every second child has a parasitic infection, whereas a quarter have two infections. Improving preventive measures, like access to safe water, improved food hygiene practices, and strengthening sanitation routines and facilities, and routine deworming, while addressing proper personal hygiene through health education for primary school children, is urgently needed. We recommend to increase awareness, strengthening public health prioritization, and conducting further studies on interventions and their reach to reduce the burden of intestinal parasitic infections among primary school children, with a particular focus on lower grades.

## Funding

This study was supported by The Norwegian aid development north–south (59360).

## Disclosure

The funders had no role in study design, data collection and analysis, decision to publish, or preparation of the manuscript.

## Ethics Statement

Ethical clearance was obtained from Hawassa University, Ethiopia (ref. No. IRB/945/26, date 02/01/2026), and from the Ethical Committee of Western Norway, Norway (Ref. No. 656883, date 11/10/2023). The institutional Review Board evaluation and the subsequent conduct of this research were guided by the Declaration of Helsinki. Following ethical approval, support letters from the Sidama National Regional Education Bureau and the Health Bureau to respective district offices were obtained for permission and cooperation. Written informed consent was obtained from parents or caregivers of children after explaining all the benefits and risks of the study, and assent was also obtained from the children. All personal identifiers remain confidential. Children who tested positive for intestinal parasitic infections were listed and linked to health facilities to ensure they received appropriate deworming and treatment. Likewise, health education regarding parasitic infections and hygiene practices was provided to all enrolled children and their parents/caregivers after data collection.

## Conflicts of Interest

The authors declare no conflicts of interest.

## Supporting Information

Additional supporting information can be found online in the Supporting Information section.

## Supporting information


**Supporting Information 1** Table S1: Bivariable logistic regression analysis among primary school children cluster robust standard error. Table S2: Negative binomial regression model for *A. lumbricoides* fecal egg count using bootstrap standard error. Table S3: Zero‐inflated negative binomial regression model for *A. lumbricoides* fecal egg count using bootstrap standard error. Table S4: Multivariable logistic regression using cluster robust standard error. Table S5: Zero‐inflated negative binomial regression model for *A. lumbricoides* fecal egg count using cluster robust standard error.

**Supporting Information 2** STROBE checklist.

## Data Availability

The data that support the findings of this study will be available upon acceptance and reasonable request.
