# Supplementary material for: Burden of Intestinal Parasitic Infections and the Intensity of Helminth Infection Among Primary School Children in Sidama, Ethiopia: A Zero‐Inflated Negative Binomial Analysis
Source: J Parasitol Res. 2026 Oct 21;2026:2036666. doi: 10.1155/japr/2036666 (PMC13685507; doi:10.1155/japr/2036666)
Supplement: Supplementary file 1 [file JAPR-2026-2036666-s001.docx]

**S4: STROBE checklist**

|  | **Item**  **number** | **Recommendation** | Manuscript section and line number |
| --- | --- | --- | --- |
| Title and Abstract | 1 | (a) Indicate the study's design with a commonly used term in the title or the abstract | Title: line number 3 |
|  |  | (b) Provide in the abstract an informative and balanced summary of what was done and what was found | Abstract: pages:2  Line number: 17 to 41 |
| Introduction | | |  |
| Background/rationale | 2 | Explain the scientific background and rationale for the investigation being reported | Introduction Pages: 3 and 4  Line number: 43 to 80 |
| Objectives | 3 | State specific objectives, including any pre specified hypotheses | Aim Page: 4  Lines number: 77 to 80 |
| Methods | | |  |
| Study design | 4 | Present key elements of study design early in the paper | Method Page:5  Line number: 85 to 86 |
| Setting | 5 | Describe the setting, locations, and relevant dates, including periods of recruitment, exposure, follow-up, and data collection | Method Page: 5  Line number 81 to 93 |
| Participants | 6 | (a) Cohort study- Give the eligibility criteria, and the sources and methods of selection of participants. Describe methods of follow-up | NA |
|  |  | Case-control study- Give the eligibility criteria, and the sources and methods of case ascertainment and control selection. Give the rationale for the choice of cases and controls | NA |
|  |  | Cross-sectional study- Give the eligibility criteria, and the sources and methods of selection of participants | Eligibility: Pages: 5  Line number: 97 to 101  Method of selection: Page 6  Line number: 115 to 116 |
|  |  | (b) Cohort study-For matched studies, give matching criteria and number of exposed and unexposed | NA |
|  |  | Case-control study-For matched studies, give matching criteria and the number of controls per case | NA |
| Variables | 7 | Clearly define all outcomes, exposures, predictors, potential confounders, and effect modifiers. Give diagnostic criteria, if applicable | Outcome: page 6  Lines number: 124 to 127  Interview: page 132 to 142  Physical examination: page 7 line number 143 to 146  Laboratory procedures: Page 7  Lines number: 147 to160 |
|  | 8^a^ | For each variable of interest, give sources of data and details of methods of assessment (measurement). | Methods: Data collection and measurements: page 6 to 7 Lines: 129 to 160 |
|  |  | Describe comparability of assessment methods if there is more than one group | NA |
| Bias | 9 | Describe any efforts to address potential sources of bias | Methods data quality control: page 7 to 8  Lines number: 161 to 174 |
| Study size | 10 | Explain how the study size was arrived at | Methods sample size: page 5 to 6  Line number: 105 to 123 |
| Quantitative variables | 11 | Explain how quantitative variables were handled in the analyses. If applicable, describe which groupings were chosen, and why | Methods Data management and analysis: page 8 to 9  Line number: 175 to 200 |
| Statistical methods | 12 | (a) Describe all statistical methods, including those used to control for confounding | Methods data management and analysis: page 8 to 9  Line number: 195 to 200 and 221 to 225 |
|  |  | (b) Describe any methods used to examine subgroups and interactions | Methods data management and analysis: page 9 Line: 201 and 207 |
|  |  | (c) Explain how missing data were addressed |  |
|  |  | (d) Cohort study-If applicable, explain how loss to follow-up was addressed | NA |
|  |  | Cross-sectional study-If applicable, describe analytical methods taking account of sampling strategy |  |
|  |  | (e) Describe any sensitivity analyses | NA |
| Results | | |  |
| Participants | 13^a^ | (a) Report the numbers of individuals at each stage of the study e.g., numbers potentially eligible, examined for eligibility, confirmed eligible, included in the study, completing follow-up, and analyzed | Result: Page 11  Line number:238 to 240 |
|  |  | (b) Give reasons for non-participation at each stage | Result: page 11 |
|  |  | (c) Consider use of a flow diagram |  |
| Descriptive data | 14^a^ | (a) Give characteristics of study participants (e.g., demographic, clinical, social) and information on exposures and potential confounders | Result: page 10 to 11  Line number: 236 to 320 |
|  |  | (b) Indicate the number of participants with missing data for each variable of interest | Result page 11 and 12 table one and two |
|  |  | (c) Cohort study-Summarise follow-up time (e.g., average and total amount) |  |
| Outcome data | 15^a^ | Cohort study-Report numbers of outcome events or summary measures over time | NA |
|  |  | Case-control study-Report numbers in each exposure category, or summary measures of exposure | NA |
|  |  | Cross-sectional study-Report numbers of outcome events or summary measures | Result: page 14 to 15  247 to 293 |
| Main results | 16 | (a) Give unadjusted estimates and, if applicable, confounder-adjusted estimates and their precision (e.g., 95% confidence interval). Make clear which confounders were adjusted for and why they were included | Unadjusted: supporting information page 25 Table: S1-S5 |
|  |  | (b) Report category boundaries when continuous variables were categorized | Method: page 9  line number: 200 to 206 |
|  |  | (c) If relevant, consider translating estimates of relative risk into absolute risk for a meaningful time period |  |
| Other analysis | 17 | Report other analyses done- e.g., analyses of subgroups and interactions, and sensitivity analyses | Data analysis: page 9  Line number:207 and 234 |
| Discussion | | |  |
| Key results | 18 | Summarise key results with reference to study objectives | Discussion: page 20 to 23  Lines: 322 to 416 |
| Limitations | 19 | Discuss limitations of the study, taking into account sources of potential bias or imprecision.  Discuss both direction and magnitude of any potential bias. | Discussion: Page 23  Lines:417 to 438 |
| Interpretation | 20 | Give a cautious overall interpretation of results considering objectives, limitations, multiplicity of analyses, results from similar studies, and other relevant evidence. | Conclusion: page 24  Lines: 439 to 447 |
| Generalizability | 21 | Discuss the generalizability (external validity) of the study results | Conclusion: page 29 |
| Other information | | |  |
| Funding | 22 | Give the source of funding and the role of the funders for the present study and, if applicable, for the original study on which the present article is based | Funding: page 25  Line number:468 to 471 |

^a^ Give such information separately for cases and controls in case-control studies, and, if applicable, for exposed and unexposed groups in cohort and cross-sectional studies.
