# Supplementary material for: Burden of Intestinal Parasitic Infections and the Intensity of Helminth Infection Among Primary School Children in Sidama, Ethiopia: A Zero‐Inflated Negative Binomial Analysis
Source: J Parasitol Res. 2026 Oct 21;2026:2036666. doi: 10.1155/japr/2036666 (PMC13685507; doi:10.1155/japr/2036666)
Supplement: Supplementary file 2 [file JAPR-2026-2036666-s002.docx]

**Supplementary**

**S1 Table1:** Bivariable logistic regression analysis using cluster robust standard error

**S2 Table 2:** Negative binomial regression model for *A. lumbricoides* fecal egg count using bootstrap standard error

**S3 Table 3:** Zero-inflated negative binomial regression model for *A. lumbricoides* fecal egg count using bootstrap standard error

**S4** **Table 4:** Multivariable logistic regression using cluster robust standard error.

**S5 Table 5:** Zero-inflated negative binomial regression model for *A. lumbricoides* fecal egg count using cluster robust standard error.

**S1 Table:** Bivariable logistic regression analysis

| **Variables** | **Option** | **Intestinal parasitic infections** | | **COR (95% CI )** | **p-value** |
| --- | --- | --- | --- | --- | --- |
|  |  | **Positive**  **n = 348** | **Negative**  **n= 274** |  |  |
| Sex | Male | 203 (58.3) | 131 (47.8) | 1.5 (1.2, 1.9) | 0.001 |
|  | Female | 145 (41.7) | 143 (52.2) | 1 |  |
| Age |  |  |  | 1.0 (0.7, 1.4) | 0.880 |
| Grade | One | 201 (57.8) | 123 (44.9) | 1.7 (1.2, 2.4) | 0.004 |
|  | Two | 147 (42.2) | 151 (55.1) | 1 |  |
| Educational status of mothers | Primary+ | 245 (70.8) | 212 (78.2) | 0.7 (0.5, 1.0) | 0.075 |
|  | No formal education | 101 (29.2) | 59 (21.8) | 1 |  |
| Educational status of fathers | Primary+ | 273 (82.2) | 234 (88.6) | 0.6 (0.4, 0.9) | 0.014 |
|  | No formal education | 59 (17.0) | 30 (11.0) | 1 |  |
| Occupation of the mothers | Housewife | 319 (92.2) | 256 (94.5) | 0.7 (0.5, 1.0) | 0.069 |
|  | Merchant or others | 27 (7.8) | 15 (5.5) | 1 |  |
| Occupation of the father | Other than farmer | 117 (35.2) | 54 (20.6) | 2.1 (1.4, 3.2) | 0.001 |
|  | Farmer | 215 (64.8) | 210 (79.6) | 1 |  |
| Wealth index | Poor | 133 (38.2) | 77 (28.1) | 1.5 (0.7, 3.1) | 0.299 |
|  | Medium | 156 (44.8) | 112 (40.9) | 1.5 (0.9, 2.4) | 0.091 |
|  | Least poor | 63 (18.1) | 68 (24.8) | 1 |  |
| Family size | ≥ 5 | 258 (74.1) | 206 (75.2) | 0.9 (0.6, 1.4) | 0.783 |
|  | < 5 | 90 (25.9) | 68 (24.8) | 1 |  |
| Walking barefoot | Yes | 52 (14.9) | 21 (7.7) | 2.1 (1.0, 4.6) | 0.057 |
|  | No | 296 (85.1) | 253 (92.3) | 1 |  |
| Child’s nail condition | Dirty and cut nails | 88 (25.6) | 58 (21.3) | 1.5 (0.8, 3.3) | 0.211 |
|  | Clean and uncut nails | 14 (4.1) | 20 (7.4) | 0.7 (0.5, 1.1) | 0.168 |
|  | Dirty and uncut nails | 205 (59.6) | 155 (57.0) | 1.3 (0.7, 2.9) | 0.381 |
|  | Clean and cut nails | 37 (10.7) | 39 (14.3) | 1 |  |
| Illness history two weeks prior to data collection | Yes | 70 (20.1) | 39 (14.2) | 1.5 (1.0, 2.2) | 0.037 |
|  | No | 278 (79.9) | 235 (85.8) | 1 |  |
| Deworming in the last 6 month | No | 73 (21.0) | 55 (20.1) | 1.1 (0.6, 1.9) | 0.848 |
|  | Yes | 275 (79.0) | 219 (79.9) | 1 |  |
| People per sleeping room | ≥ 5 | 294 (84.5) | 243 (88.7) | 1.4 (0.6, 3.2) | 0.381 |
|  | < 5 | 54 (15.5) | 31 (11.3) | 1 |  |
| Separate cooking room | No | 177 (50.9) | 115 (42.0) | 1.4 (1.0, 2.0) | 0.024 |
|  | Yes | 171 (49.1) | 159 (58.0) | 1 |  |
| Domestic animals | Yes | 103 (29.6) | 89 (32.5) | 0.9 (0.6, 1.4) | 0.545 |
|  | No | 245 (70.4) | 185 (67.5) | 1 |  |
| Type of latrine | Unimproved | 325 (93.4) | 244 (89.0) | 1.7 (0.9, 3.3) | 0.091 |
|  | Improved | 23 (6.6) | 30 (11.0) | 1 |  |

**S2 Table 2.** Negative binomial regression model for *A. lumbricoides* fecal egg count

| Variables | Option | Negative binomial | | |
| --- | --- | --- | --- | --- |
|  |  | Robust standard error | Infection intensity  IRR (95% CI) | p-value |
| Sex | Male | 0.4 | 1.5 (0.9, 2.6) | 0.110 |
|  | Female | 1 | 1 |  |
| Grade | One | 0.5 | 1.8 (1.0, 3.1)* | 0.037 |
|  | Two | 1 | 1 |  |
| Educational status of mothers | Primary+ | 0.1 | 0.5 (0.3, 0.8)* | 0.011 |
|  | No formal education | 1 | 1 |  |
| Occupation of fathers | Other than farmer | 0.7 | 2.2 (1.3, 4.0)* | 0.004 |
|  | Farmer | 1 | 1 |  |
| Wealth index | Poor | 0.3 | 0.7 (0.3, 1.8) | 0.499 |
|  | Medium | 0.3 | 1.3 (0.8, 2.0) | 0.213 |
|  | Least poor | 1 | 1 |  |
| Walking barefoot | Yes | 0.6 | 1.3 (0.6, 3.2) | 0.504 |
|  | No | 1 | 1 |  |
| Illness history two weeks prior to data collection | Yes | 0.6 | 1.6 (0.8, 3.3) | 0.170 |
|  | No | 1 | 1 |  |
| People per sleeping room | ≥ 5 | 0.5 | 1.3 (0.6, 2.8) | 0.505 |
|  | < 5 | 1 | 1 |  |
| Separate cooking room | No | 0.4 | 0.9 (0.4, 2.1) | 0.829 |
|  | Yes | 1 | 1 |  |
| Alpha |  | 4.0 | 15.0 (8.8, 25.3) |  |

*= p-value < 0.05, bootstrap report

**S3 Table 3:** Zero-inflated negative binomial regression model for *A. lumbricoides* fecal egg count

| Variables | Option | Zero-inflated negative binomial | | | | | | |
| --- | --- | --- | --- | --- | --- | --- | --- | --- |
|  |  | Negative binomial | | | | Zero-inflated | | |
|  |  | bootstrap standard error | Infection intensity  IRR (95% CI) | p-value | | bootstrap standard error | Infection probability  AOR (95% CI) | p-value |
| Sex | Male | 0.2 | 1.2 (0.9, 1.6) | 0.205 | | 0.1 | 0.6 (0.5, 0.8)* | 0.001 |
|  | Female | 1 | 1 |  | |  | 1 |  |
| Grade | One | 0.3 | 1.4 (1.0, 2.3) | 0.055 | | 0.1 | 0.7 (0.6, 0.9)* | 0.004 |
|  | Two | 1 | 1 |  | |  | 1 |  |
| Educational status of mothers | Primary+ | 0.1 | 0.5 (0.4, 0.8)* | 0.006 | | 0.2 | 1.4 (1.0, 2.1) | 0.086 |
|  | No formal education | 1 | 1 |  | |  | 1 |  |
| Occupation of fathers | Other than farmer | 0.2 | 1.7 (1.4, 2.1)* | 0.001 | | 0.2 | 0.6 (0.4, 0.8)* | 0.006 |
|  | Farmer | 1 | 1 |  | |  | 1 |  |
| Wealth index | Poor | 0.2 | 0.8 (0.5, 1.2) | 0.223 | | 0.5 | 1.0 (0.4, 2.4) | 0.962 |
|  | Medium | 0.2 | 1.1 (0.8, 1.5) | 0.424 | | 0.3 | 0.8 (0.5, 1.3) | 0.393 |
|  | Least poor | 1 | 1 |  | |  | 1 |  |
| Walking barefoot | Yes | 0.2 | 1.2 (0.9, 1.7) | 0.302 | | 0.4 | 0.7 (0.3, 1.4) | 0.289 |
|  | No | 1 | 1 |  | |  | 1 |  |
| Illness history two weeks prior to data collection | Yes | 0.3 | 1.5 (0.9, 2.3) | 0.097 | | 0.3 | 0.9 (0.5, 1.3) | 0.518 |
|  | No | 1 | 1 |  | |  | 1 |  |
| People per sleeping room | ≥ 5 | 0.3 | 1.3 (0.8, 2.1) | 0.253 | | 0.4 | 0.9 (0.5, 1.9) | 0.858 |
|  | < 5 | 1 | 1 |  | |  | 1 |  |
| Separate cooking room | No | 0.3 | 0.9 (0.5, 1.8) | 0.791 | | 0.2 | 1.0 (0.7, 1.3) | 0.836 |
|  | Yes | 1 | 1 |  | |  | 1 |  |
| Alpha |  | 0.3 |  | |  | | 3.1 (2.5, 3.8) |  |

*= p-value < 0.05, bootstrap report

**Table 4.** Multivariable logistic regression using cluster roubust standard error.

| Variables | Options | Intestinal parasitic infections | | COR (95% CI) | AOR (95% CI) |
| --- | --- | --- | --- | --- | --- |
|  |  | Positive  n = 348 | Negative  n = 274 |  |  |
| Sex | Male | 203 (58.3) | 131 (47.8) | 1.5 (1.2, 1.9) | 1.5 (1.2, 1.9)* |
|  | Female | 145 (41.7) | 143 (52.2) | 1 | 1 |
| Grade | One | 201 (57.8) | 123 (44.9) | 1.7 (1.2, 2.4) | 1.5 (1.1, 2.1)* |
|  | Two | 147 (42.2) | 151 (55.1) | 1 | 1 |
| Educational status of mothers | Primary+ | 245 (70.8) | 212 (78.2) | 0.7 (0.4, 1.1) | 0.7 (0.5, 1.1) |
|  | No formal education | 101 (29.2) | 59 (21.8) | 1 | 1 |
| Occupation of the father | Other than farmer | 117 (35.2) | 54 (20.4) | 2.1 (1.4, 3.2) | 2.1 (1.4, 2.8)* |
|  | Farmer | 215 (64.8) | 210 (79.6) | 1 | 1 |
| Wealth index | Poor | 133 (38.2) | 77 (28.1) | 1.5 (0.7, 3.1) | 1.0 (0.5, 2.0) |
|  | Medium | 156 (44.8) | 112 (40.9) | 1.5 (0.9, 2.4) | 1.2 (0.8, 1.7) |
|  | Least poor | 63 (18.1) | 68 (24.8) | 1 | 1 |
| Walking barefoot | Yes | 52 (14.9) | 21 (7.7) | 2.1 (1.0, 4.6) | 1.4 (0.8, 2.4) |
|  | No | 296 (85.1) | 253 (92.3) | 1 | 1 |
| Illness history two weeks prior to data collection | Yes | 70 (20.1) | 39 (14.2) | 1.5 (1.0, 2.2) | 1.2 (0.8, 1.9) |
|  | No | 278 (79.9) | 235 (85.8) | 1 | 1 |
| Separate cooking room | No | 177 (50.9) | 115 (42.0) | 1.4 (1.0, 2.0) | 1.2 (0.9, 1.7) |
|  | Yes | 171 (49.1) | 159 (58.0) | 1 | 1 |

Note: COR = crude odds ratio, AOR= adjusted odds ratio, * = p-value < 0.05, Educational status primary+ = (primary, secondary and above), Occupation other than farmer = (Daily laborer, government employer/non-governmental organization, merchant, or other)

Table 5: Zero-inflated negative binomial regression model for *A. lumbricoides* fecal egg count using cluster robust standard error

| Variables | Options | Zero-inflated negative binomial | |
| --- | --- | --- | --- |
|  |  | Negative binomial | Zero-inflated |
|  |  | Infection intensity  IRR (95% CI) | Infection probability  AOR (95% CI) |
| Sex | Male | 1.2 (0.9, 1.6) | 0.6 (0.5, 0.8)* |
|  | Female | 1 | 1 |
| Grade | One | 1.4 (1.0, 2.3) | 0.7 (0.6, 0.9)* |
|  | Two | 1 | 1 |
| Educational status of mothers | Primary+ | 0.5 (0.4, 0.8)* | 1.4 (1.0, 2.1) |
|  | No formal education | 1 | 1 |
| Occupation of fathers | Other than farmer | 1.7 (1.4, 2.1)* | 0.6 (0.4, 0.8)* |
|  | Farmer | 1 | 1 |
| Wealth index | Poor | 0.8 (0.5, 1.2) | 1.0 (0.4, 2.4) |
|  | Medium | 1.1 (0.8, 1.5) | 0.8 (0.5, 1.3) |
|  | Least poor | 1 | 1 |
| Walking barefoot | Yes | 1.2 (0.9, 1.7) | 0.7 (0.3, 1.4) |
|  | No | 1 | 1 |
| Illness history two weeks prior to data collection | Yes | 1.5 (0.9, 2.3) | 0.9 (0.5, 1.3) |
|  | No | 1 | 1 |
| People per sleeping room | ≥ 5 | 1.3 (0.8, 2.1) | 0.9 (0.5, 1.9) |
|  | < 5 | 1 | 1 |
| Separate cooking room | No | 0.9 (0.5, 1.8) | 1.0 (0.7, 1.3) |
|  | Yes | 1 | 1 |
